# Current concepts regarding the HTLV-1 receptor complex

**DOI:** 10.1186/1742-4690-7-99

**Published:** 2010-11-29

**Authors:** David Ghez, Yves Lepelletier, Kathryn S Jones, Claudine Pique, Olivier Hermine

**Affiliations:** 1 CNRS UMR8147, Universite Rene Descartes, Paris 5, 161 Rue de Sèvres, 75743 Paris Cedex 15, France; 2 Service d'Hématologie, Institut Gustave Roussy, 39 rue Camille Desmoulins 94805 Villejuif, France; 3 SAIC-Frederick, Inc., NCI-Frederick, Frederick, MD, USA; 4 Institut Cochin, Université Paris Descartes, CNRS (UMR 8104), Paris, France; 5 Inserm, U1016, Paris, France; 6 Service d'Hématologie Adulte, Hôpital Necker, 161 Rue de Sèvres, 75743 Paris CEDEX 15, France

## Abstract

The identity of the Human T lymphotropic Virus type 1 (HTLV-1) receptor remained an unsolved puzzle for two decades, until the recent demonstration that three molecules, Glucose Transporter 1, Neuropilin-1 and Heparan Sulfate Proteoglycans are involved in HTLV-1 binding and entry. Despite these advances, several questions remain unanswered, including the precise role of each of these molecules during virus entry. In light of the most recent data, we propose a model of the HTLV-1 receptor complex and discuss its potential impact on HTLV-1 infection.

## Introduction

Since its identification in 1979, the Human T-Lymphotropic Virus type 1 (HTLV-1) has been the subject of extensive research. Despite abundant experimental data, several unsolved mysteries still surround HTLV-1. Amongst those, the identity of the HTLV-1 cellular receptor has eluded scientists for over two decades. Since 2003, data from several independent groups have shed light on the identity of the molecules that appear to be directly involved in the process of HTLV-1 entry. These include Glucose Transporter 1 (GLUT1) [1,2], Neuropilin-1 (NRP-1) [3-5] and Heparan Sulfate Proteoglycans (HSPG) [6,7]. Despite these advances, our current knowledge of their precise roles during HTLV-1 entry remains limited. In particular, little is known about interaction between these molecules, including whether they form a multimolecular complex similar to that of the well-studied Human Immunodeficiency Virus type 1 (HIV-1) receptor. In this review, we will summarize the data pertaining to HTLV-1 entry molecules, propose a hypothetical model of the HTLV-1 receptor and discuss its possible impact on the pathobiology of HTLV-1 infection.

## The HTLV-1 Receptor Enigma

### HTLV-1 entry

It is widely believed that HTLV-1 enters cells in a manner similar to that of other retroviruses including the well-studied HIV-1. HTLV-1 infection of target cells is believed to require the two virally encoded envelope glycoproteins (Env), the surface subunit (SU) gp46 and the transmembrane subunit (TM) gp21, generated from the cleavage of a polyprecursor (gp61) in the Golgi apparatus [8]. As has previously been shown for the SU (gp120) and TM (gp41) of HIV-1 [9], the SU and TM of HTLV-1 are believed to function successively during the entry process. The gp46/SU, which does not contain a transmembrane region, is tethered to the cell surface through interactions with the gp21, and is involved in direct interactions with the cell surface receptors. Like the HIV-1 gp41, the gp21 of HTLV-1 contains a transmembrane region and a N-terminal hydrophobic fusion peptide that plays a crucial role in the fusion of the viral and cellular membrane during the final steps of HTLV-1 entry [10,11]. Earlier work showed that both the presence of certain mutations in the *env *gene and antibodies directed against gp46/SU partially or totally abolished HTLV-1 infection *in vitro *[12,13]. Infection was also reduced by exposure of target cells to a soluble recombinant protein containing soluble full-length SU, indicating that it could compete with the SU on the surface of the virus for the binding to yet unknown molecules at the cell surface [14]. Altogether, these experiments confirmed that the two Env glycoproteins play crucial role during HTLV-1 entry and indicated that, as for other retroviruses, this process depends on the expression of cellular receptor(s) at the surface of target cells. Interaction between gp46/SU and its cellular receptor(s) induces a conformational change that unmasks the fusion domain within the TM, allowing fusion between the viral and cellular membranes [15].

### The search for the HTLV receptor

Experimental demonstration that specific molecules function as a virus receptor often is a challenge [16]. This is illustrated by the fact that there are a great number of viruses, including certain retroviruses, for which no receptors have been identified. In addition, for certain viruses, it has been shown that more than one molecule may function as entry receptors. Some viruses use different receptors on different cell types, while others require the presence of both molecules on a given target cell to facilitate entry. In the case of HTLV-1, several peculiar features of the virus and its receptor(s) have considerably hampered research. First, free HTLV-1 virions are poorly infectious for most cell types and efficient infection of T cells requires a cellular contact [17]. Secondly, infected lymphocytes produce a limited amount of viral particles, amongst which 1 out of 10^5 ^are actually infectious [18]. Lastly, the fact that molecules capable of binding and allowing HTLV-1 entry are expressed on nearly all available established cell lines has prevented the use of classical strategies such as the screening of a cDNA bank in receptor-negative cells.

### *In vivo *versus *in vitro *entry tropism: the paradox of the HTLV-1 receptor

Virus tropism, the ability of a virus to replicate in particular cells, depends on interactions between viral components and cellular factors at each step of the viral cycle from the initial entry to the ultimate release and transmission of virions. Here, we will focus on the cellular factors that allow HTLV-1 entry, which is determined by the distribution of the receptor molecule(s).

#### In vivo entry tropism

Although the main targets of HTLV-1 are CD4^+ ^T cells [19], the virus has been found in other cell types *in vivo *including as CD8^+ ^T cells [20], monocytes and B cells [21], macrophages [22], dendritic cells (DC) [23,24] and endothelial cells [25]. *In vivo*, the only known targets of virus-induced transformation are CD4^+ ^CD45RO^+ ^memory T cells [26]. Recently, it has been hypothesized that natural regulatory T cells (Tregs) can be infected. This was based on the fact that HTLV-1 infected T cells and Tregs have a strikingly close phenotype: CD25+, (which is a direct consequence of HTLV-1 Tax synthesis [27]), GITR+ and FoxP3+ [28,29]. If true, this could partly explain the frequent immune dysregulation observed in HTLV-1-infected individuals. However, studies from other laboratories suggest that Tregs are not infected by the virus [30,31]. This is consistent with other work showing potent suppressive activity of HTLV-1-infected or transformed T cells in only a portion of HTLV-1-infected patients [32,33]. This could be due to the impairment of FoxP3 function by the HTLV-1 Tax protein, as shown by Jacobson's group [34]. In contrast, it has been suggested that HTLV-1 may have an indirect effect on Tregs since Bangham's group recently reported that the frequency of uninfected functional Tregs is abnormally high in HTLV-1-infected individuals [35], which may account as well for the immunosuppression associated with HTLV-1 infection.

#### In vitro entry tropism

In striking contrast to the limited number of cell-types in which HTLV-1 is detected *in vivo*, molecules capable of supporting the initial steps in infection, binding and entry, appear to be expressed on nearly all established cell lines. As discussed below, this is consistent with the fact that molecules in the receptor complex are up-regulated on most transformed cells. Moreover, the HTLV-1 receptor complex is present on cell lines from nearly all known vertebrate species [36]: most available established cell lines are able to form multinucleated giant cells (syncytia) when cultured with Env expressing cells, a phenomenon that is dependent on Env/Receptor interactions [37]. Further evidence that HTLV-1 receptors are widely distributed comes from observations that HTLV-1 Env-pseudotyped particles can infect a number of established cell lines [38]. More recently, binding studies with soluble, full-length HTLV-1 SU (SU-Fc) has not only confirmed the results obtained with infection and fusion experiments but also showed that a wider range of established cell lines expressed molecules capable of specifically binding the SU protein [1]. Work from the Brighty laboratory found that the SU-Fc protein could bind to a vast number of vertebrate cell lines including some that were originally thought to be receptor negative due to their resistance to Env-mediated cell to cell fusion or infection [39]. This study identified one cell line, the drosophila cell line S2, that lacked molecules capable of specifically binding HTLV SU on the cell surface. Unfortunately, these cells could not be used to identify HTLV-1 receptors, since they have post-entry blocks to HTLV-1 and other retroviruses.

### Properties of the HTLV-1 receptor: indirect evidence

Although its ubiquitous nature considerably complicated its identification, several properties of the HTLV-1 receptor, in particular its expression pattern on primary cells, were characterized using indirect approaches. In 2003, two independent groups generated soluble SU proteins by generating recombinant SU-Ig-Fc fusion proteins containing either the full-length SU [22] or the N-terminal portion of SU (Receptor Binding Domain, see section 3.1) [40]. Their findings showed that the receptor was not present at the surface of resting CD4^+ ^T cells but was rapidly upregulated upon activation. The receptor was also absent on naive T cells isolated from cord blood but could similarly be upregulated after stimulation by interleukin-7. Finally, it was reported that the SU-Fc could inhibit a mixed lymphocyte reaction [22]. This last property suggested that one of the molecules involved in the HTLV-1 receptor was a member of the immune synapse. Surface expression of the receptor was found to be not solely dependent on T cell activation. It could also be upregulated upon exposure to TGF-β, a potent negative regulator of the immune response [41]. The pattern of surface expression following exposure to TGF-β, as determined from binding of the soluble SU, was very similar to that observed after activation with PHA/IL-2, although with slightly slower kinetics. It depended on the TGF-β classical signalling pathway as it was abolished in the presence of Smad inhibitors. TGF-β not only triggered receptor expression at the cell surface but also increased the titer of lentiviral vectors pseudotyped with HTLV-1 Env *in vitro*, demonstrating that molecules capable of both binding and fusion were expressed [41]. TGF-β-treated T cells remained in a resting state, demonstrating that T cell activation *per se *was not required for receptor expression. This might constitute a strategy for the virus to increase its infectivity [42] as HTLV-1-infected CD4^+ ^T cells are known to abundantly secrete TGF-β.

### Candidate receptors whose role was ruled out

Over the years, several molecules were proposed as candidate HTLV-1 receptors. It was proposed that the HTLV-1 receptor is encoded by chromosome 17q23.2-23.5, which was questioned in later studies [38,43]. Most of the candidate receptors were identified as antigens against which specific antibodies could inhibit Env-mediated cell fusion [43-46]. It was later shown that some antibodies may inhibit this process through non-specific protein crowding, rather than directly competing for binding, making the results difficult to interpret [47]. Adhesion molecules, which strengthen and stabilize the cell-to-cell contact, can also augment cell fusion in a non-specific manner [48]. Most importantly, none of these molecules had demonstrated one fundamental property of the receptor, that is, the ability to bind the HTLV-1 SU. In contrast, as it could bind the 197-216 region of the SU, the heat shock cognate protein HSC70 was proposed as a receptor [49]. However, later studies showed that, while HSC70 modulates HTLV-1-induced syncytia formation, it was dispensable for HTLV-1 infection [50], ruling out that this molecule was an entry receptor. The tetraspanin CD82 was also shown to bind the HTLV-1 Env protein but was excluded as an entry receptor on the fact that its overexpression inhibited, rather than enhanced, syncytia formation and HTLV-1 transmission [51].

## Not one but Three Receptor Molecules: GLUT1, HSPG and NRP-1

### The glucose transporter GLUT1

It was not until 2003 that work from Sitbon and Battini's laboratory identified a candidate that matched all the prerequisites to be a HTLV-1 receptor [2]. Furthermore, this group determined that this molecule interacted with both HTLV-1 and the related virus HTLV-2, believed to use a common entry receptor. After noticing that overexpression of either the HTLV-1 or HTLV-2 Env in cells prevented acidification of the culture medium, the authors hypothesized that the HTLV-1 receptor might be related to the proton-dependent lactate production. Based on their previous identification of the receptor binding domain (RBD) of HTLV-1 or HTLV-2, these authors further reported that the overexpression of either the H1-RBD (first 215 residues of the HTLV-1 SU) or the H2-RBD (178 residues of the HTLV-2) [40,52,53] altered glucose metabolism, which was consistent with the frequent use of metabolite transporters as entry receptors by retroviruses [16]. They focused on the ubiquitous glucose transporter 1 (GLUT1), which is upregulated in activated T cells, and found that overexpression of GLUT-1 in cells increased the level of binding of both the H1- or H2-RBD. In the absence of available GLUT1-negative cell lines, they used different approaches, in particular small interfering RNA (siRNA), to demonstrate that GLUT1 played a role in HTLV-1 entry. Down regulation of GLUT1 by siRNA both inhibited the binding of the H1- or H2-RBD and infection by retroviral vectors pseudotyped with either the HTLV-1 or -2 Env. Both were restored after cotransfection of GLUT1 but not GLUT3, a related glucose transporter. They later extended their findings by that identifying a specific residue in the HTLV-1 SU (Y114) of the H1-RBD that appeared critical to the interaction [52], which interacted with the 6^th ^extracellular loop (ECL6) of GLUT1 [54].

Further data from other laboratories confirmed that GLUT1 plays a role in HTLV-1 entry. TGF-β, which had previously been shown to upregulate molecules capable of binding HTLV SU, induces GLUT1 at the surface of T cells [41]. Overexpression of GLUT1 in the bovine MDBK cell line, which is relatively resistant to HTLV-1 infection, increases two-fold the infectious titre of a HTLV-1 Env pseudotyped lentivirus vector [55]. A similar result was obtained in the NIH3T3 cell line, which is also poorly susceptible to infection by HTLV-1 pseudotyped vectors [1]. Antibodies raised against GLUT1 ECL1 (GLUT-IgY) block both Env-mediated fusion and infection. Furthermore, blocking interactions with GLUT1 ECL1, either by replacing the GLUT1 ECL1 with that of GLUT3, or by blocking with ECL1-derived peptides, inhibits HTLV-1-induced cell fusion, suggesting that ECL1 is critical for the receptor activity of GLUT1 [1]. This confirmed earlier data showing that, although only the ECL6 was required for the binding of the H1-RBD to GLUT1, infection by HTLV-2-pseudotyped viruses also required residues located in ECL1 and ECL5 of this molecule [54]. This indicates that GLUT1 contains multiple functional determinants: those required for interactions with the RBD portion of the HTLV SU, located in ECL6, and others required for interactions of the entire Env, consisting of the full-length SU protein and the TM protein, located in ECL1 and ECL5. Thus, current data indicate that GLUT1 plays a role in HTLV-1 entry.

However, several studies suggested that the identification of GLUT1 was not the end of the story. The astrocytoma/glioblastoma U87 cell line, which expresses very low levels of GLUT-1 due to a transdominant negative mutation in one of the alleles of GLUT1 (GLUT-1 D5), is easily infected by HTLV-pseudotyped viruses[3,56]. Moreover, blocking interactions with GLUT1 by either siRNA-mediated down-regulation, or by incubating with GLUT-IgY blocking antibodies, has no effect on the level of infection with HTLV-Env-pseudotyped virus or HTLV Env-mediated fusion or infection in this cell line, suggesting that molecules other than GLUT1 might be involved [56]. An earlier study reported that overexpression of GLUT1 in a cell line (COS-7) increased cell-cell transmission, but did not increase the level of binding of the SU-Fc protein [57]. Similarly, more recent studies using clones of the U87 cell line that express different levels of GLUT1 have observed that the level of cell surface GLUT1 correlates with the titer of HTLV-1 Env pseudotyped viruses, but not with SU-Fc binding [58]. All these findings suggested that molecules other than GLUT1 are also involved in HTLV-1 entry, especially at the binding step.

### Heparan Sulfate Proteoglycans modulate HTLV-1 attachment and entry

Molecules of the HSPG family are composed of a core protein associated with one or more sulphated polysaccharide side chains called heparan sulfate glycosaminoglycans. Because of their highly negative charge, HSPG bind through electrostatic interactions to a plethora of proteins including growth factors and their receptors, chemokines, cytokines and numerous proteins of the extracellular matrix or the plasma, thereby playing a major role in mammalian physiology [59]. Pathogens, including many viruses, have been shown to hijack HSPG [60]. HSPG generally enhance infection by facilitating the attachment of the particles on target cells and/or allowing their clustering at the cell surface before specific interactions between viral proteins and their receptors that lead to fusion. For example, prior to interaction of the HIV SU (gp120) with CD4, the initial attachment of the virus to target cells involves specific interactions between gp120 and cellular HSPG [61]. Rarely, a specific role of HSPG in the fusion process has been observed, in particular with Herpes Simplex virus [62].

The important role of HSPG in mediating attachment and entry has also been demonstrated for HTLV-1. Enzymatic removal of HSPG or inhibition of electrostatic interactions with dextran sulfate decreases the binding of full length soluble SU, Env-mediated syncytium formation and infection with pseudotyped viruses [7]. These initial findings, obtained in non-lymphoid cell lines expressing high levels of HSPG, were later confirmed in CD4^+ ^T cells in Jones and Ruscetti's laboratory [6]. Although HSPG are barely detectable on quiescent T cells [63], they are rapidly upregulated upon activation. In CD4^+ ^T cells, HSPG augment both the binding of the SU-Fc and the infectious titer of pseudotyped viruses [6]. The importance of HSPG was suggested by studies showing that removing HSPG reduced binding and entry of HTLV-1 virus into CD4+ T cells and dendritic cells [4,6] and significantly reduced the level of HTLV-1 infection.

Other recent studies suggest another role for HSPG during cell-cell transmission of the virus. It was discovered that HTLV-1 virions are stored outside the cell, within a protective microenvironment enriched for specific components of the extracellular matrix, including lectins and HSPGs [64]. Following contact between T cells, these structures are rapidly transferred from infected to uninfected cells, ultimately resulting in infection of the target cell. This suggests that HSPG could play a broader role in HTLV-1 transmission by facilitating both the transfer of newly-formed virions from an infected T cells and the subsequent entry into the target T cell. The galactose-binding lectin Galactin-1, which has been shown to increase HTLV-1-Env mediated infection [65], might also have a similar function.

Interestingly, HSPG are important for infection with HTLV-1 but not HTLV-2 [66], showing that these two viruses, initially considered to share the same receptor [67], may share some but not all molecular determinants for entry. It has previously been reported that HTLV-1 SU has the ability to bind HSPG; this ability maps to the C-terminal region of HTLV-1 SU, located downstream the RBD and the proline rich region [66]. Interestingly, the Green laboratory has previously reported that the preferential tropism of HTLV-1 and HTLV-2 to transform CD4^+ ^T cells or CD8^+ ^T cells, respectively, is governed by Env [68]. The difference in the requirement of HTLV-1 and HTLV-2 two SU for HSPG might explain the different tropisms of the two retroviruses. Indeed, CD4^+ ^T cells express a high level of HSPG and a lower level of GLUT1 whereas CD8^+ ^T cells express a high level of GLUT1 but very few HSPG. In this model, the differences between HTLV-1 and HTLV-2 would be similar to what has been described in CD4-dependent and independent HIV-2 or SIV strains, which differ in their requirements for the binding receptor (CD4) but share similar fusion receptors [69].

### The third player: Neuropilin-1

In 2006, the Hermine and Pique laboratories showed that Neuropilin-1 (NRP-1) also displayed properties expected for a HTLV-1 receptor [5]. This hypothesis was confirmed in a recent study [3]. NRP-1 is a 130 kDa single membrane spanning glycoprotein which acts as the co-receptor for Semaphorin 3a and VEGFA_165_. NRP-1 was initially identified as a critically important factor in embryonic neuron guidance, and later shown to be a key player in the regulation of angiogenesis. In addition, Romeo and Hermine's laboratories were the first to demonstrate that NRP-1 is also involved in the regulation of the immune response [70]. NRP-1 is highly conserved amongst vertebrate species but there are no NRP-1 homologs in insects [71]. NRP-1 is mainly expressed in T cells and DC, which are targets of HTLV-1 *in vivo*. NRP-1 is highly expressed on plasmacytoid DC (pDC), which can be infected by cell-free virus *in vitro *[72]. Endothelial cells, in which HTLV-1 proviral DNA has been detected *in vivo *[25], also express NRP-1 [73]. NRP-1 is absent on resting T cells but is rapidly upregulated following activation [72,74]. In contrast with its relatively limited expression *in vivo*, NRP-1 is found in many tumor cells [73] and hence is expressed on an extremely broad range of established cell lines. Finally, NRP-1 plays a role in cytoskeletal rearrangement, a phenomenon that has been shown to be important for transmission of different retroviruses including HTLV-1 [75].

Ghez et al. reported that NRP-1 was able to directly interact with the HTLV-1 and -2 SU [5]. This interaction appeared functionally relevant since NRP-1 overexpression enhanced syncytium formation and the titer of HTLV-1 or -2 Env pseudotyped viruses whereas siRNA-mediated NRP-1 downmodulation had the opposite effect [5]. A strong polarization of NRP-1 and Env on either side of the interface between an infected cell and a target T cell was observed using confocal microscopy. This phenomenon was also observed with GLUT1, though GLUT1 was also found to colocalize at the membrane junction of two uninfected T cells, thus in an Env-independent manner. Both polarization and co-localization of the three molecules were particularly intense at regions where partial membrane fusion was taking place. It was also observed that GLUT1 and NRP-1 could form intracytoplasmic complexes in transfected cells, an association that was greatly enhanced in the presence of Env [5].

### A new understanding of HTLV-1 tropism

The identification of three new molecules involved in the process of HTLV-1 entry allows one to revisit the HTLV-1 tropism paradox mentioned above. While HSPG usage appears to distinguish HTLV-1 and HTLV-2, their ubiquitous expression cannot explain the limited tropism of HTLV-1 *in vivo*. This is also the case of GLUT1, whose expression is ubiquitous as well. In contrast, the distinct pattern of NRP-1 expression *in vivo *and *in vitro *may explain the disparity between the *in vivo *and *in vitro *tropisms. In primary cells, NRP-1 is mainly expressed on endothelial cells, activated CD4^+ ^T cells and DC, all of which have been observed to be infected *in vivo*. However, NRP-1 overexpression upon cell transformation renders it nearly ubiquitous among established cell lines. Moreover, the *nrp-1 *gene is strongly conserved between mammalian species and has no homolog in insects. These findings strongly favor the notion that the *in vivo *HTLV-1 tropism is mainly dictated by NRP-1 expression.

## At Last, Several Receptors or a Receptor Complex?

Thus, after more than 20 years, three different molecules have proven to be important for HTLV-1 entry. It seems clear that HSPG, as for nearly all other viruses, is involved in binding but not in fusion. The properties displayed by both GLUT1 and NRP-1, i.e. binding to the HTLV-1 SU and modulation of infection, are consistent with those expected for a receptor but, as mentioned above, many questions on their exact function remain unsolved. In particular, it is not clear whether GLUT1 and NRP-1 cooperate to promote HTLV-1 entry. We will review very recent data that have started to clarify the respective roles of HSPG, NRP-1 and GLUT1 in HTLV-1 infection.

### GLUT1 and NRP-1 bind distinct regions of the HTLV-1 SU

As was hypothesized previously, there is now definitive evidence that GLUT1 and NRP-1 bind to distinct regions of SU. Kim *et al. *determined that the minimal RBD is located within the first 183 amino terminal residues of the HTLV-1 SU [52]. Mutation of the tyrosine residue 114 completely abolished the binding of H1-RBD, suggesting it plays a critical role. However, the results of binding studies using RBD should be interpreted with caution, as they might not truly represent what occurs with the full length SU or the SU/TM complex in the native Env. Several lines of evidence suggest that this may be the case. For example, over expression of GLUT1 strongly increased binding of the H1-RBD [2], but has little impact on the binding of full-length HTLV-1 SU [57]. It is thus possible that shortening the SU modifies its quaternary structure and mimics a conformational change resulting in an "active" form capable of binding directly to GLUT1, possibly by exposing the tyrosine residue 114 [52]. It is tempting to speculate that the conformational change allowing the association to GLUT1 might be dependent on the initial SU binding to NRP-1. This would be analogous to what occurs for HIV, where SU (gp120) binding to CD4 induces a conformational change that allows binding to the co receptors, which facilitates fusion.

The HSPG-interacting SU region is located outside the GLUT1 and NRP-1 binding sites, and thus should not decrease its affinity to the other two molecules; this interpretation is consistent with the data showing that increasing cell surface levels of HSPG increases SU binding to receptor-expressing cells [6,7]. Blocking the ability of SU to interact with HSPG, either by enzymatic removal or incubation with a peptide mimicking the HSPG-binding domain of VEGFA_165 _(exon-7 peptide), decreased the DC-mediated transfer of HTLV-1 to CD4+ T cells by 50%. Moreover, blocking both HSPG and NRP-1 interactions decreased HTLV-1 infection by 80% [4,72]. Thus, HSPG are important for the efficiency of HTLV-1 infection, likely by increasing and stabilizing the initial SU binding to NRP-1, a phenomenon already described for gp120 and CD4 [76].

In 2009, Lambert *et al. *found that the binding of the HTLV-1 SU to NRP-1 involved two distinct types of interaction: an indirect interaction mediated by HSPG and a direct interaction mediated by the amino-acids 90-94 of the SU [4]. Moreover, they identified a region of SU (aa 90-94) that is homologous in both sequence and function to the exon 8 domain of the NRP-1 ligand VEGFA_165_, [77]. This demonstrates that the SU binds to NRP-1 using molecular mimicry of VEGFA_165_. Interestingly, the aa 90-94 motif is conserved in the SU proteins of HTLV-2, HTLV-3 and related Simian T-Lymphotropic Viruses but not other retroviruses [4] and is the target of neutralizing antibodies [78]. Strikingly, Kim *et al. *showed that mutating either aa 94 (R) or 114 (Y), which inhibits direct interaction with NRP-1 or GLUT1 respectively, is equally efficient in preventing interference due to overexpression of the H1-RBD in target cells [52]. This reinforces the notion that the direct interaction of SU with both NRP-1 and GLUT1 is required for HTLV-1 entry.

### Towards a hypothetical model of the HTLV-1 receptor

**Figure 1:**
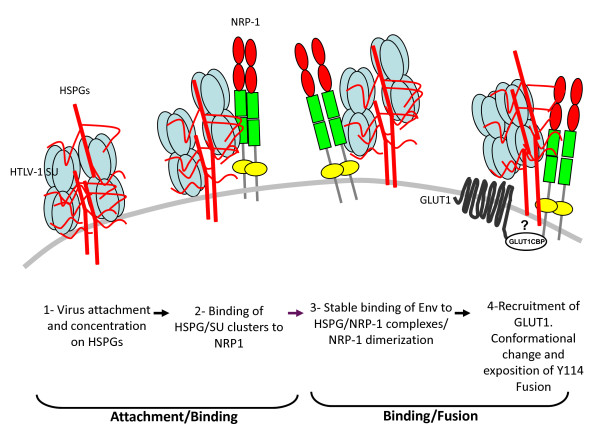
**Hypothetical model of the HTLV receptor complex and HTLV entry**: 1- HSPG interaction with the SU allow the initial attachment and concentration of the virions at the cell surface. 2- HSPG facilitates the recruitment of the SU to NRP-1. 3- Using molecular mimicry of VEGFA_165_, the SU region 90-94 interacts with the b domain of NRP-1, an interaction that is stabilized by HSPG. 4- SU-NRP-1 interaction triggers a conformational change within the SU, allowing exposure of the tyrosine 114 that is critical for binding to GLUT1. Interaction between GLUT1 and the SU triggers a conformational change allowing the unmasking of the TM fusion peptide (not depicted). The small adaptor protein GLUT1CBP might form a link between NRP1 and GLUT1 and stabilize the receptor complex.

In light of these data, it is possible to draw a model for HTLV-1 entry based on an analogy with HIV CD4/CXCR4 or CCR5 receptor complexes, and what is known about interactions of NRP-1 with its ligand VEGFA_165_, HSPG, and receptor (Figure 1). During the initial binding phase, the SU would interact with HSPG via its C-terminal portion, allowing its attachment to the cell surface (Figure 1, step 1). This would enhance the local concentration of SU, and thus increase the probability of interactions with NRP-1. This notion is consistent with observations that removal of cell surface HSPG dramatically reduces the level of binding of the SU or virions to CD4^+ ^T cells [6], the titer of pseudotyped virus, and the infection of CD4^+ ^T cells and DC. HSPG/SU complexes would then interact with the b domain of NRP-1, through both HSPG/NRP-1 interactions and direct SU/NRP-1 binding mediated by the 90-94 region of the SU (step 2). This binding to NRP-1 could trigger a conformational change within the SU, exposing the region necessary for GLUT1 binding including the critical Y114. Finally, the interaction of the SU with GLUT1 could trigger the fusion process necessary to HTLV-1 entry (step 3). In this model, NRP1, with the help of HSPG, is the primary binding receptor, while GLUT1 is the fusion receptor. This is consistent with the formation of NRP-1/Env/GLUT1 complexes and with the fact that GLUT1 over expression does not increase the level of HTLV-1 SU binding. This model applies to both primary CD4^+ ^T cells and DC [4,72]. Recent data showing that HTLV-1 entry into primary astrocytes requires both NRP-1 and GLUT1 [3] suggest that this view is true for these cells as well.

Whether this model is relevant for HTLV-1 entry in other types of cells remains to be investigated. A recent paper from Alkhatib's group showed that GLUT1, but not NRP-1, was involved in HTLV-1 entry into Hela cells [3]. Moreover, the same group was the first to show that HTLV-1 can efficiently enter into U87 cells, in which the surface level of GLUT1 is very low [56]. Low level of GLUT1 at the cell surface could be sufficient to promote fusion if the density of surface NRP1 is high, which is the case of the U87 cell line [3]. Indeed, it was shown that T cells expressing very low levels of CCR5 but high levels of CD4 were still competent for HIV envelope-mediated membrane fusion [79]. Alternatively, NRP-1 could function on certain cell types as both the binding and fusion receptor, as suggested for CHO cells [3]. Finally, one cannot exclude the possibility that, like HIV, HTLV-1 uses different fusion receptors on different cell types and other unidentified molecules could serve as fusion receptors for HTLV-1.

Other evidence supporting a NRP-1/GLUT1- complex comes from reports that a small intracytoplasmic protein called GLUT1CBP or PSD-95/Dlg/ZO-1 can bind both NRP-1 and GLUT1 via its PDZ domain [80,81]. Moreover, syndecans, the main members of the HSPG family, also contain a PDZ-binding motif at their C-terminus of their cytoplasmic domain [82]. Deletion of the PDZ-binding motif of GLUT1 was shown to preclude GLUT1 clustering at the plasma membrane [83]. GLUT1CBP could therefore act as a "bridge" to stabilize the entire tripartite receptor complex. It could also provide cues for cytoskeletal polarisation, since it can associate with several cytoskeletal proteins [80] (Figure 1, step 3). Altogether, this model not only reconciles nearly all of the recent data obtained on the HTLV-1 receptor but also appears to explain the *in vivo *tropism of the virus better than the GLUT1-only hypothesis.

## Perspectives

Given its role in the immune system, the discovery of NRP-1 as an entry molecule for HTLV-1 opens interesting perspectives on viral transmission and the physiology of HTLV-1 related diseases.

### Targeting dendritic cells and disturbing the immune response

Plasmacytoid DC (pDC), and to a lesser extent myeloid dendritic cells (myDC), constitutively express high levels of NRP-1, which is involved in the regulation of DC-T cell interactions at the immune synapse [70]. Shaking the commonly accepted idea that HTLV-1 could only be transmitted via cell to cell contact, it was recently found that free HTLV-1 virions could efficiently infect DC [72]. The contact of HTLV-1-infected DC with CD4^+ ^T cells upregulates NRP-1 and HSPG within an hour followed by transmission of HTLV-1 to T cells and productive infection. This phenomenon appears to be dependent on both NRP-1 and HSPG expression on the target T cells [72]. Viral transmission may occur in *cis *(from virus produced from infected DCs) but also in *trans *(prior to infection of the DC), as has been previously shown for DC-mediated HIV infection. This raises the question of whether DC (in particular pDC) could constitute a reservoir for the virus. Several laboratories have reported detecting HTLV-1 sequences in DC *in vivo *[84]. It would be interesting to determine whether, in contrast to T cells, an active replication cycle takes place in pDC *in vivo*.

Targeting DC and interfering with NRP-1 signalling might have several other consequences on viral infection. HTLV-1 infection results in a depletion of myDC as well as pDC and an impairment of their IFNα-producing capacity [85]. Furthermore, the number of pDC is negatively correlated to the proviral load, suggesting a certain degree of impairment of the antiviral response. It is still not known whether this pDC decrease is due to true depletion or, as in HIV infection, to their migration into lymphoid tissues. Recent *in vitro *data demonstrated that cell-free HTLV-1 generates a pDC innate immune response by an induction of costimulatory molecules, production of massive levels of IFN-α, and rapid expression of the apoptotic ligand TRAIL. The role of NRP-1 in this process remains to be clarified. Furthermore, HTLV-1-stimulated pDC was shown to induce apoptosis of CD4^+ ^T cells expressing DR5, transforming pDC into Interferon-producing Killer pDC (IKpDC) [86]. Interaction of HTLV-1 SU binding and NRP-1 in infected cells might by itself disturb DC-T cells interactions, and thus impair the immune response. This is consistent with previous reports that soluble HTLV-1 SU has an inhibitory effect on a mixed lymphocyte reaction [22]. This phenomenon might be of particular significance during primary infection when there is an active transcription of viral products including Env, which could thus dampen the primary antiviral response. Alternatively, IKpDC may reduce spreading of infection by killing infected T cells and allowing the emergence of few infected T cell clones in the infected host. Finally, infection of immature myDC might disturb T cell homeostasis and lead to an increased risk of autoimmune phenomena.

### Potential effects of NRP-1 ligands

#### VEGFA_165_

Data clearly show that VEGFA_165 _is a potent competitor of HTLV-1 SU binding and HTLV-1 infection of primary T cells and DC *in vitro *[4,72]. VEGFA_165 _secretion by HTLV-1-infected cells [87] might explain the phenomenon of receptor interference. By preventing the generalised spreading of the virus, it might also explain the peculiar course of HTLV-1 infection in which a limited number of T cell clones are infected following primary infection. Because it increases NRP-1 expression at the surface of target cells [88], VEGFA_165 _might not only have a negative effect. Sherer *et al*. showed in an elegant model of MLV infection that target cells projected filopodiae towards MLV infected cells [89]. These expansions, which the authors termed viral cytonemes, create an intercellular bridge that allows the virus to "slide" towards its target cell and bind to its receptor. Endothelial cells, which have been found to be infected *in vivo *[25] and coexpress NRP-1 and VEGFR2, are able to project similar cytoplasmic expansions, or filopodiae, towards VEGFA_165 _[90]. Although the relevance of this phenomenon in other retroviral infections remains to be studied, a similar mechanism driven by the interaction between NRP-1 and either VEGFA_165 _or Env itself might be envisaged with HTLV-1.

In ATLL it has been shown that VEGFA_165 _production may participate in cell growth and angiogenesis [91,92]. At this stage of the disease, the NRP-1/VEGFA_165 _interaction may occur because the virus Env is no longer expressed in vivo.

#### Semaphorin-3a

Another major NRP-1 ligand, sema3a, could also play a role in the regulation of HTLV-1 infection. Polarization of the cytoskeleton is a critical determinant of transmission in a number of retroviruses including HIV-1 and HTLV-1 [75]. Although Tax has been shown to accumulate at the cell-cell junction and be involved in the microtubule reorganization [84], little is known about the precise mechanisms responsible for this phenomenon. In the central nervous system, sema3a blocks F-actin polymerization, which results in the repulsion of the axon growth cone [93]. In the immune system, sema3a-NRP-1 interactions inhibit the cytoskeletal polarization following the contact between a DC and a T cell [74]. This process may provide the negative feedback cues to disorganize the immune synapse, allowing T cells to migrate from DC. In HTLV-1 infection, sema3a could play a negative role by inhibiting the cytoskeletal rearrangement that is necessary for HTLV-1 entry. Thus, interaction between the Env and NRP-1 may prevent the action of Sema3A on the activated T cell target, thus allowing HTLV-1 infection.

## Conclusion

The recent discovery that GLUT1, NRP-1 and HSPG are involved in HTLV-1 entry sheds new light on HTLV-1 infection and the physiopathology of HTLV-1-related diseases. The demonstration that HTLV-1 uses molecules involved in the immune response and can infect dendritic cells more efficiently than CD4^+ ^T cells provides fascinating perspectives for future research. Further work is clearly needed to better define the precise role of the elements of this "ménage à trois" and its consequence on infected individuals. A better understanding of the effect of HTLV-1 infection on the immune system could lead to the development of alternative strategies in the treatment of HTLV-1-related diseases early in the course of the infection or even at a later stage, as in ATLL.

## Competing interests

The authors declare that they have no competing interests.

## Authors' contributions

OH and DG designed the manuscript. DG wrote the manuscript. YL, KSJ, CP revised and added new information to the manuscript. OH coordinated the work and finalized the manuscript with DG.
